# Genetic background modifies phenotypic and transcriptional responses in a *C. elegans* model of α-synuclein toxicity

**DOI:** 10.1186/s12864-019-5597-1

**Published:** 2019-03-20

**Authors:** Yiru A. Wang, Basten L. Snoek, Mark G. Sterken, Joost A. G. Riksen, Jana J. Stastna, Jan E. Kammenga, Simon C. Harvey

**Affiliations:** 1 Biomolecular Research Group, School of Human and Life Sciences, Canterbury Christ Church University, North Holmes Road, Canterbury, CT1 1QU UK; 2 Laboratory of Nematology, Wageningen University, 6708 PB Wageningen, The Netherlands; 3 Theoretical Biology and Bioinformatics, Utrecht University, Utrecht, The Netherlands

**Keywords:** Natural variation, Gene expression profile, Protein aggregation, α-Synuclein, Genetic background, *Caenorhabditis elegans*

## Abstract

**Background:**

Accumulation of protein aggregates are a major hallmark of progressive neurodegenerative disorders such as Parkinson’s disease and Alzheimer’s disease. Transgenic *Caenorhabditis elegans* nematodes expressing the human synaptic protein α-synuclein in body wall muscle show inclusions of aggregated protein, which affects similar genetic pathways as in humans. It is not however known how the effects of α-synuclein expression in *C. elegans* differs among genetic backgrounds. Here, we compared gene expression patterns and investigated the phenotypic consequences of transgenic α-synuclein expression in five different *C. elegans* genetic backgrounds.

**Results:**

Transcriptome analysis indicates that α-synuclein expression effects pathways associated with nutrient storage, lipid transportation and ion exchange and that effects vary depending on the genetic background. These gene expression changes predict that a range of phenotypes will be affected by α-synuclein expression. We confirm this, showing that α-synuclein expression delayed development, reduced lifespan, increased rate of matricidal hatching, and slows pharyngeal pumping. Critically, these phenotypic effects depend on the genetic background and coincide with the core changes in gene expression.

**Conclusions:**

Together, our results show genotype-specific effects and core alterations in both gene expression and in phenotype in response to α-synuclein expression. We conclude that the effects of α-synuclein expression are substantially modified by the genetic background, illustrating that genetic background needs to be considered in *C. elegans* models of neurodegenerative disease.

**Electronic supplementary material:**

The online version of this article (10.1186/s12864-019-5597-1) contains supplementary material, which is available to authorized users.

## Background

Dementia is a growing global problem affecting large numbers of people. The most common causes of dementia are associated with neurodegeneration, such as that resulting from Alzheimer’s disease (AD) and Parkinson’s disease (PD) [1–3]. These neurodegenerative dementias share neuroanatomical and biochemical similarities and result from protein misfolding. A major difficulty in determining the mechanisms that produce neurodegenerative dementia is that the underlying cellular-level pathology varies greatly among patients (reviewed in [4, 5]). These differences in pathology allow for genome-wide association studies (GWAS) of PD patients, identifying genetic risk-variants and demonstrate the complex genetic architecture of neurodegenerative dementia [6–8].

Model organisms, such as the nematode *Caenorhabditis elegans*, are of great value for studying neurodegenerative diseases [9–13]. This is due to their experimental tractability and to the broad and general conservation of genetic pathways across species. Importantly, *C. elegans* allows for sophisticated genome-wide genetic screens, which are much less complex than similar studies on mammals. In *C. elegans*, most of these screens have sought to identify loci that represent potential candidate diagnostic and therapeutic targets, or to understand the underlying pathological processes. For example, analysis of transgenic *C. elegans* expressing the human amyloid-β peptide – the main component of the amyloid plaques found in AD – linked toxicity to insulin/insulin-like growth factor (IGF), dietary restriction and the heat shock response [9, 10, 12, 14–16]. Similarly for PD, analysis of worms expressing α-synuclein identified associations with ageing and insulin-like signalling [17] and with autophagy and lysosomal function [18].

Most *C. elegans* studies are however limited to the canonical Bristol N2 genetic background, and therefore do not provide insight into how genetic variation between individuals might affect disease-associated traits. This is a major issue as it is clear, both specifically in *C. elegans* (e.g. [19–22]) and more generally in other systems [23–25], that the phenotype of any given mutation, transgene or allele can vary depending on the genetic background [26]. For *C. elegans*, natural genetic variation is known to result in extensive phenotypic variation (e.g. [27, 28], and see [29, 30] for reviews for older studies) and differentially affects both the proteome [31, 32] and transcriptome [22, 31, 33–37]. Therefore, only studying mutational effects in a single genetic background biases our understanding of disease phenotypes, and represents a missed opportunity to elucidate disease mechanisms.

Exemplifying this pattern of a reliance on a single genetic background, only one study has, to date, looked at protein misfolding disease in multiple wild isolate genetic backgrounds of *C. elegans* [38]. Crucially, this showed that natural genetic variation can uncouple different phenotypic effects of polyglutamine (polyQ40) expression [38]. This strongly suggests that similar important variation between genetic backgrounds will be found for other protein misfolding diseases. Given that for protein misfolding diseases the nature of the modifying alleles segregating within human populations remain largely elusive [39], the experimental tractability of *C. elegans* in combination with genetic variation makes the species an excellent system in which to address this issue.

In *C. elegans*, expression of an α-synuclein and yellow fluorescent protein (YFP) fusion in the body wall muscle results in an age-dependent accumulation of inclusions [17]. These inclusions of α-synuclein form aggregates in aging worms that are similar to the pathological inclusions seen in humans with PD [17]. This therefore represents an appropriate model of α-synuclein toxicity [40]. To investigate the effect of genetic background on the consequences of α-synuclein expression, we have created introgression lines (ILs) containing this α-synuclein and YFP transgene in the background of four wild isolates of *C. elegans*. Our analyses of these new ILs, and of α-synuclein in an N2 genetic background, identify both general and genotype-specific changes in gene expression. These changes predict a range of phenotypic effects that we then experimentally confirm. Importantly, given the reliance on N2 in *C. elegans* research, we show both that some effects are N2-specific and that for other phenotypes the analysis of other genetic backgrounds uncovers substantial variation not seen in N2. Our approach therefore evaluates how genetic background conditions transgene effect(s), and specifically illustrates how the consequences of ectopic overexpression of α-synuclein depends on genetic background.

## Results

### Introgression line construction and validation

We introgressed the *pkIs2386* transgene [unc-54p::α-synuclein::YFP + unc-119(+)] from NL5901 [17], which has an N2 genetic background, into the genetically divergent wild isolates JU1511, JU1926, JU1931, and JU1941 (see [35] for information on genetic distance between these lines). The *pkIs2386* transgene results in α-synuclein expression in the body wall musculature and the vulval muscles. After back-crossing and selfing, four new ILs were obtained, SCH1511, SCH1926, SCH1931, and SCH1941, with for example, SCH1511 containing the transgene in a JU1511 background. In combination with N2 and NL5901 as controls, we were therefore able to investigate the phenotypic and genomic effects of α-synuclein in five genetic backgrounds (N2, JU1511, JU1926, JU1931, and JU1941). An additional IL, SCH4856, was subsequently created in which the transgene has been introgressed into a CB4856 background. SCH4856 was used to test for the effects of N2 alleles (see below).

We sought to identify the site of the introgression and to determine how much of the N2 genome surrounding the transgene had also been introgressed into the wild isolates. PCR-based genotyping located the transgene in chromosome IV, indicating that the homozygous introgressions in the new ILs spanned between 4.2 and 13.2Mb of chromosome IV (Additional file 1). By using the set of genetic markers from Volkers et al. [35], we identified significantly differential expressed genes on chromosome IV of the α-synuclein lines, and also identified an additional introgression on chromosome V in SCH1931 (Additional file 2). PCR-based genotyping together with the transcriptomic analysis indicated a consistent genomic location for the α-synuclein transgene in each of the genetic backgrounds, but did not allow detection of the precise introgression boundaries.

### Transcriptome effects of the α-synuclein introgression depend on the genetic background

**Fig. 1:**
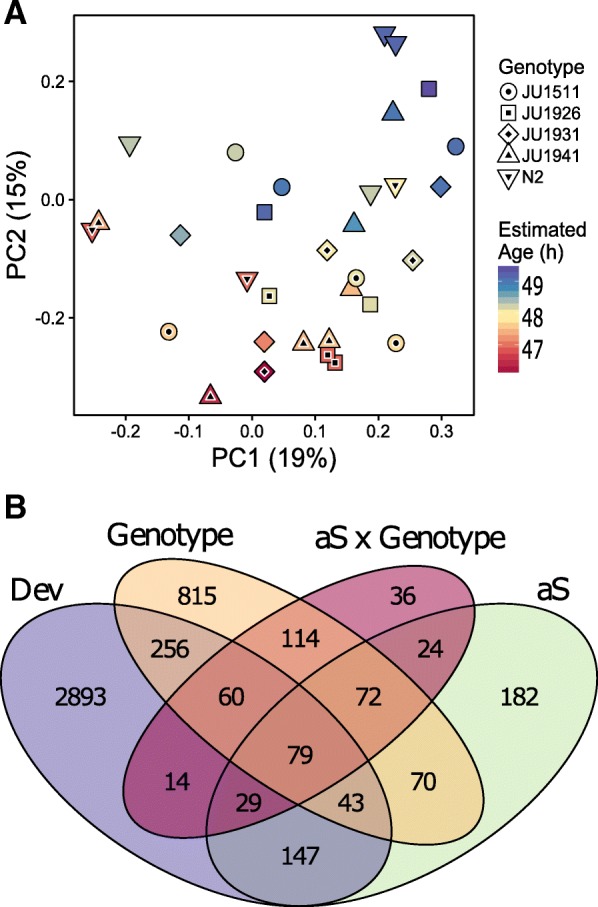
Gene expression varies with genetic background in *C. elegans* ILs constitutively expressing α-synuclein. **a** Principal component analysis on gene expression differences, with genotypes represented by shape and α-synuclein (aS) expressing ILs indicated with a black point. Estimated age difference is shown by the colour gradient. Here, developmental age was determined based on the expression levels of class I age responsive genes from Snoek et al. [41], which show a linear increase in expression during development between 46 and 54 h. This indicated a maximum developmental difference of a 2.5 h between genotypes. **b** Venn diagram of differentially expressed genes that are specific to aS, Genotype, Age and aS x Genotype including both induced and repressed genes

The effects of α-synuclein expression on gene expression was measured in five different genetic backgrounds, with three biological replicates per genotype (see Additional file 3 and ArrayExpress accession E-MTAB-6960 for details). Analysis of genome-wide transcriptional changes in the α-synuclein expressing worms indicated that there were differences in developmental rate between the ILs. Variation between ILs in developmental rate was also observed during IL construction. We therefore estimated the age of the samples by their gene expression profile (as in [41, 42]) and used principal component analysis (PCA) on genome-wide expression levels to investigate differences between isolates and the effects of α-synuclein. This analysis revealed separation between the wild isolates and their corresponding transgenic ILs (Fig. 1a). Although genotypes were more scattered on these first two PCA axes, this indicated that developmental delay and reduced lifespan were associated with the introgressed α-synuclein transgene (Fig. 1a). A large part of the global gene expression differences are therefore explained by slower development in the α-synuclein lines. Hence, many of the genes identified as responding to α-synuclein in the different backgrounds were likely to be associated with their differential age. Using a threshold of -log10(*p*) > 3.4 (FDR < 0.05) we found 3521 genes affected by Age, 1509 affected by Genotype, 646 by α-synuclein and 428 by an interaction between Genotype and α-synuclein (Fig. 1b, Additional file 4). Genes affected by α-synuclein or the interaction between α-synuclein and age were most often also affected by genotype. The overlap among these four factors yielded 78 genes highly specific for α-synuclein relative to both Age and Genotype (Additional file 4). This showed that both genotype-specific and universal (genotype independent) changes in gene expression were induced by α-synuclein expression, i.e. we identified a core set of genes of which the expression was altered in all genetic backgrounds and others that were genotype-specific. Strikingly, many genes were not expressed and/or regulated in the same way in wild isolate genetic backgrounds compared with N2. These data therefore indicate that the transcriptome effects of the α-synuclein introgression depend on the genetic background.

### Gene expression enrichment analysis

Gene ontology analysis (GO) of differentially expressed genes revealed the molecular, cellular, and biological processes affected by development, α-synuclein and genetic background (Table 1, with full results in Additional file 4). Genes that change expression in response to genetic background were enriched for genes involved in the innate immune response and oxidation-reduction process (Additional file 4) as was previously found by [35]. Widespread changes in genes related to muscle function were observed (Table 1), an expected response given the changes in cellular environment induced by expression of α-synuclein in the body wall muscle. This analysis also identified changes in genes involved in pharyngeal pumping (Table 1).

**Table 1:** The top-20 enriched GO-terms with 5 or more genes with an α-synuclein effect

GO_ID	Description	All Genes	α-synuclein Genes	Type	*p*-value	adjusted *p*
GO:0005576	extracellular region	329	54	CC	3.99E-06	8.29E-04
GO:0030246	carbohydrate binding	288	48	MF	8.12E-06	1.13E-03
GO:0005861	troponin complex	8	5	CC	1.21E-05	1.26E-03
GO:0055120	striated muscle dense body	93	21	CC	1.77E-05	1.48E-03
GO:0045087	innate immune response	300	47	BP	5.16E-05	3.06E-03
GO:0010332	response to gamma radiation	10	5	BP	7.77E-05	3.59E-03
GO:0006869	lipid transport	26	8	BP	2.74E-04	1.12E-02
GO:0098869	cellular oxidant detoxification	52	12	BP	5.12E-04	1.29E-02
GO:0030017	sarcomere	22	7	CC	4.10E-04	1.29E-02
GO:0006936	muscle contraction	17	6	BP	3.93E-04	1.29E-02
GO:0016810	hydrolase activity acting on carbon-nitrogen (but not peptide) bonds	13	5	MF	5.02E-04	1.29E-02
GO:0071949	FAD binding	18	6	MF	5.94E-04	1.37E-02
GO:0008652	cellular amino acid biosynthetic process	20	6	BP	1.23E-03	2.57E-02
GO:0043050	pharyngeal pumping	16	5	BP	1.85E-03	2.96E-02
GO:0016787	hydrolase activity	818	96	MF	2.24E-03	3.45E-02
GO:0016311	dephosphorylation	63	12	BP	3.36E-03	3.49E-02
GO:0030170	pyridoxal phosphate binding	48	10	MF	2.88E-03	3.49E-02
GO:0031430	M band	28	7	CC	2.46E-03	3.49E-02
GO:0003993	acid phosphatase activity	28	7	MF	2.46E-03	3.49E-02
GO:0099132	ATP hydrolysis coupled cation transmembrane transport	23	6	BP	3.08E-03	3.49E-02

*CC* cellular component, *MF* molecular function, *BP* biological process

As expected there were also changes in various pathways associated with cellular stress responses (Table 1), and protein homeostasis (Additional file 4). Taking the effect of both α-synuclein and age into consideration, genes associated with metabolic processes, transporters of ions and lipids, and kinase activities for ATP were enriched (Additional file 4).

### Phenotypic effects of α-synuclein expression vary among genetic backgrounds

#### Expression of α-synuclein slows development in some genetic backgrounds

**Fig. 2:**
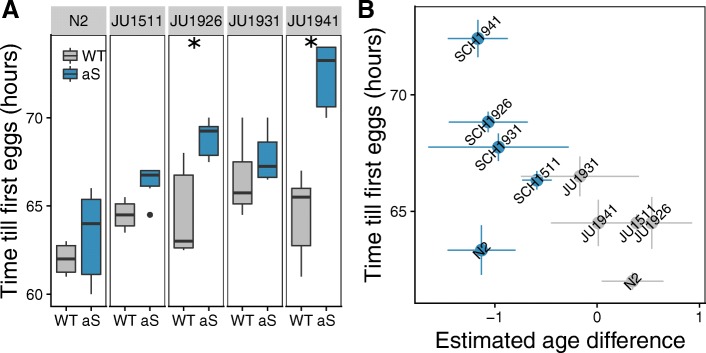
Development time varies with genetic background in *C. elegans* ILs constitutively expressing α-synuclein. **a** Boxplot of development measured by time until first egg-laying showing developmental delay of α-synuclein expressing lines and variation between genotypes (ANOVA: aS effect *p* < 2e-8; Genotype *p* < 2e-8; aS x Genotype Interaction *p* < 0.0002; Tukey HSD: N2 *p* = 0.96; JU1511 *p* = 0.88; JU1926 *p* < 0.008; JU1931 *p* = 0.98; JU1941 *p* < 1e-6, with significant differences between genotypes indicated by the stars). **b** Scatterplot of the relation between estimated age at transcriptomics sampling and the development time. Estimated age difference was determined based on the expression levels of class I age responsive genes from Snoek et al. [41], which show a linear increase in expression during development between 46 and 54 h. The points represent the mean and the lines indicate the standard error

Gene expression analysis indicated that α-synuclein lines were developmentally delayed. To test this directly, we scored development time, i.e. the time to the first appearance of eggs. Analysis of these data indicated that development was affected by α-synuclein (aS, *p* < 2e-8), genetic background (Genotype, *p* < 2e-8) and the interaction between α-synuclein and genetic background (aS x Genotype, *p* < 0.0002). In most α-synuclein expressing lines development was delayed compared to the corresponding wild isolate (Fig. 2a), with SCH1941 and SCH1926 showing significantly longer larval development time periods than the corresponding wild type controls (*p* < 1e-6, and *p* < 0.008 for SCH1941 and SCH1926, respectively) (Fig. 2a).

As found in the phenotypic assay, the transcriptomic samples of the α-synuclein lines were estimated to be younger than the corresponding wild isolate and so showed delayed development (Fig. 2b, ANOVA model Estimated_Age ~ aS*Genotype; aS *p* < 6e-5), which was independent of the genetic background (Genotype, *p* = 0.72) and of the interaction between α-synuclein and genetic background (aS x Genotype, *p* = 0.84). Comparing the WT and α-synuclein lines for each individual line showed that SCH1931 and JU1931 again displayed the smallest developmental difference as found in the phenotypic assay. Overall, we therefore conclude that the α-synuclein introgression had a genotype-specific impact, differentially decreasing the developmental rate in each genetic background.

#### Expression of α-synuclein decreases pharyngeal pumping rate in some genetic backgrounds

**Fig. 3:**
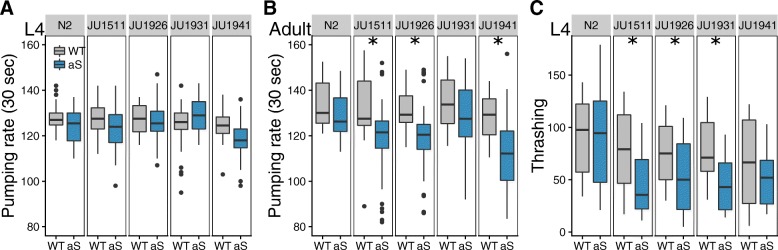
Muscle function varies with genetic background in *C. elegans* ILs constitutively expressing α-synuclein. Boxplots of the number of pharyngeal pumps over a 30 s period in worms at (**a**) 48 and (**b**) 72 h after recovery from L1 arrest. Black dots represent outliers (two times interquartile range). Analysis shows no significant effects at 48 h. At 72 h however, pharyngeal pumping is affected by α-synuclein expression, by the genetic background and by the interaction of α-synuclein expression with the genetic background (ANOVA: aS, *p* < 3e-16, Genotype, *p* < 3e-10, aS x Genotype, *p* < 0.004; Tukey HSD: N2 *p* = 0.93; JU1511 *p* < 5e-4; JU1926 *p* < 2e-5; JU1931 *p* = 0.54; JU1941 *p* < 1e-8). **c** Boxplots of the activity rates of worms in liquid, with analysis indicating that locomotion pumping is affected by α-synuclein expression, by the genetic background and by the interaction of α-synuclein expression with the genetic background (ANOVA: aS *p* < 1.953e-12, Genotype *p* < 4.4e-13, aS x Genotype *p* = 0.0040; Tukey HSD: N2 *p* = 1.00; JU1511 *p* = 2.4e-6; JU1926 *p* = 0.0016; JU1931 *p* = 1.3e-3; JU1941 *p* = 0.51). Significant differences between genotypes are indicated by stars

Given the observation of differential expression of genes with a function in pharyngeal pumping (Table 1), we measured pharyngeal pumping rate in all lines 48 and 72 h after recovery from L1 arrest (Fig. 3). No significant differences were seen at 48 h, but 72 h after recovery from L1 arrest pharyngeal pumping was affected by α-synuclein (aS, *p* < 3e-16), by the genetic background (Genotype, *p* < 3e-10) and by the interaction of α-synuclein with genetic background (aS x Genotype, *p* < 0.004; Fig. 3b). At this point, the pharyngeal pumping rate at 72 h in the α-synuclein expressing lines had slowed down compared to their corresponding wild isolate (Fig. 3b; N2 *p* = 0.93; JU1511 *p* < 5e-4; JU1926 *p* < 2e-5; JU1931 *p* = 0.54; JU1941 *p* < 1e-8). These data therefore indicated that the presence of an α-synuclein introgression had a noticeable genotype-specific impact on pharyngeal pumping rate. As pumping rate primarily reflects pharyngeal muscle activity, a tissue in which the α-synuclein transgene is not expressed, these changes reflect an indirect systemic effect of α-synuclein expression.

#### Expression of α-synuclein decreases movement in some genetic backgrounds

Given the site of α-synuclein expression and the detection of changes in the expression of genes involved in muscle structure and function (Table 1) we tested for differences between lines in movement. Here, we assayed movement of worms in liquid (Fig. 3c). These data indicate that N2 and NL5901 show similar movement rates and that the α-synuclein expressing lines expression show reduced activity in comparison to their corresponding wild isolate. These data therefore indicate that the presence of an α-synuclein introgression produces a genotype-specific impact, likely a direct consequence of α-synuclein expression in the body wall muscles.

#### Expression of α-synuclein decreases lifespan in some genetic backgrounds

**Fig. 4:**
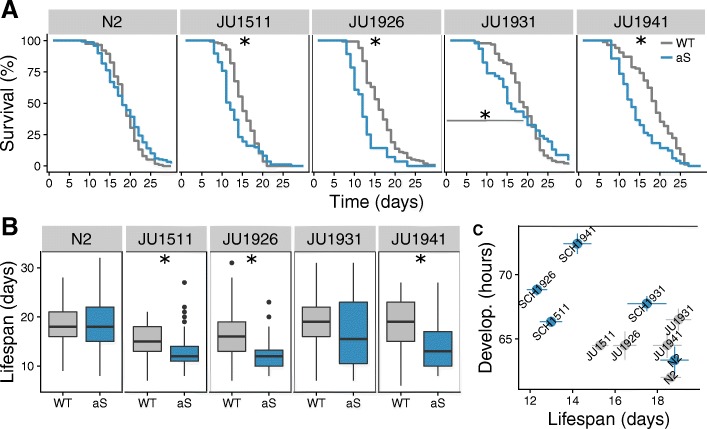
*C. elegans* ILs constitutively expressing α-synuclein exhibit significantly shorter lifespan. **a** Survival curves for wild-type (WT) and transgenic nematodes expressing α-synuclein (aS) at 20°C (log-rank test (‘survival’ package, R): N2 *p* = 0.14; JU1511 *p* < 0.003; JU1926 *p* < 4*1e-7; JU1931 *p* = 0.79 (first 20 days *p* < 0.0004); JU1941 *p* < 7e-5, with significant differences between genotypes indicated by stars). **b** Box-plot of the lifespan for WT and aS lines. Black dots denote outliers (two times interquartile range), with analysis indicating that lifespan is affected by α-synuclein expression, by the genetic background and by the interaction of α-synuclein expression with the genetic background (ANOVA: aS *p* < 3e-5; Genotype *p* < < 1e-16; aS x Genotype *p* < 0.0002; Tukey HSD: N2 *p* = 0.99; JU1511 *p* < 0.008; JU1926 *p* < 0.0004; JU1931 *p* = 0.71; JU1941 *p* < 1e-5, with significant differences between genotypes indicated by stars). **c** Relationship between lifespan and development time. The points represent the mean and the lines indicate the standard error

Given the age-related changes in pharyngeal pumping rate and enrichment of genes associated with aging and stress response pathways in response to α-synuclein expression, we hypothesised that the introgressed α-synuclein transgene could affect longevity. Therefore, we measured lifespan in all lines (Additional file 5). Comparison of N2 and NL5901 (α-synuclein in an N2 background) showed that lifespan was not affected by α-synuclein expression in the N2 genetic background (Fig. 4; N2 log-rank *p* = 0.14, mean age *p* = 0.99). However, in the other genetic backgrounds, lines containing the α-synuclein introgression displayed significantly accelerated death (Fig. 4a) and a shortened lifespan (Fig. 4b), a global effect of α-synuclein expression on animal physiology and function. These data also indicated that the α-synuclein introgression in the wild isolate genetic backgrounds resulted in increased rates of maternal hatching (bagging) (Additional file 6, Fig. 5). Given that the α-synuclein transgene is expressed in the vulval muscles this result mirrors the direct effect of α-synuclein expression on muscle function seen for thrashing (Fig. 3c). These observations indicate that α-synuclein expression lowers lifespan in some genotypes, but not others.

**Fig. 5:**
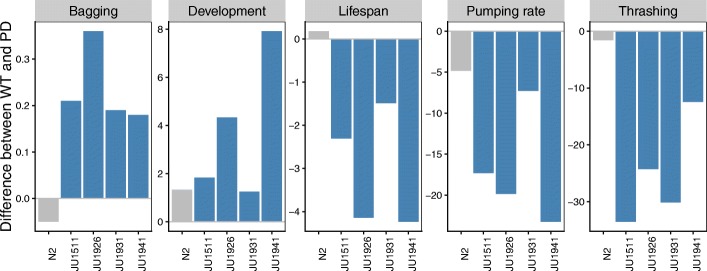
Phenotypic effects of α-synuclein expression across different genetic backgrounds. Shown are differences between wild type and α-synuclein expressing lines in bagging (% difference in matricidal hatching), in development (difference in hours from L1 arrest to first egg lay), in lifespan (difference in mean lifespan in days), in pumping rate (difference in number of pumps recorded over a 30 s period) and in trashing (activity counts in liquid recorded over a 30m period)

#### Genetic background affects the outcome of α-synuclein expression

Comparison of phenotypic effects across the genetic backgrounds tested suggests that the phenotypes associated with the α-synuclein transgene introgression vary between genotypes in a consistent manner (Fig. 5). Multiple phenotypes were affected by α-synuclein in the JU1511, JU1926, and JU1941 background, whereas they were less affected in the JU1931 and N2 backgrounds. This suggests a shared genetic component across many of the phenotypes we have observed, but does also indicate that there is genotype-specific variation, e.g. no pair of lines shows the same pattern of phenotypic effects (Fig. 5). These data also indicate that there are both local effects – e.g. bagging and trashing, where effects can be directly attributed to expression of α-synuclein in the muscles – and global effects on the whole animal.

**Fig. 6:**
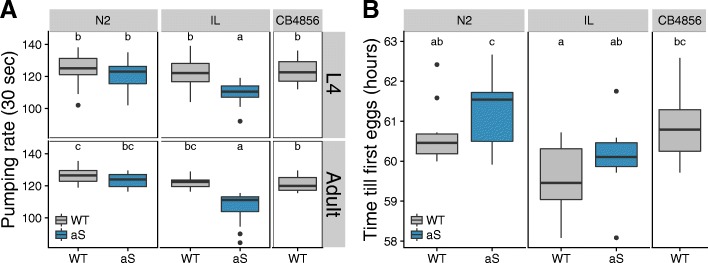
Phenotypic effects observed in a CB4856 genetic background depend on α-synuclein expression rather than N2 alleles. Here comparisons are made between N2 (N2 WT and AS) and CB4856 (CB4856 and IL AS) genetic backgrounds with and without the α-synuclein introgression and with CBN93 (IL WT), a line with an introgression of the N2 genome spanning the 3.3–12.8Mbp region on chromosome IV in an CB4856 background. **a** A box-plot of the average pumping rate in 30 s of worms 48 and 72 h after recovery after L1 arrest, and **b** Time from recovery of L1 arrest to first egg lay. The black dots represent outliers (outside two times the interquartile range). Letter codes denote lines that differ significantly (*p* < 0.05)

As alleles from different *C. elegans* backgrounds can produce a range of synthetic deleterious effects resulting in full or partially genomic incompatibilities between their genomes [43, 44], we sought to test if the N2 region alone replicated the phenotypes we observed here. We therefore introgressed the *pkIs2386* transgene into a CB4856 genetic background, generating the SCH4856 line and undertook comparisons of this line with CB4856 and CBN93, a line with an introgression of the N2 genome spanning the 3.3–12.8Mbp region on chromosome IV in an CB4856 background (Additional file 1). This control was undertaken as deleterious interactions between alleles from N2 and those from CB4856 are well characterized [43, 44]. Comparisons of pumping rate and of development time between these lines indicated that the expression of α-synuclein in SCH4856 produced effects not seen in CBN93 (Fig. 6) and that these effects mirror those seen in the other genetic backgrounds. This provides strong support for the view that the phenotypic effects we observe were a consequence of α-synuclein expression and not the introgression of the N2 region.

## Discussion

We have introgressed a transgene that results in the expression of α-synuclein in the body wall and vulval muscles into different genetic backgrounds of *C. elegans*. Analysis of these newly created α-synuclein ILs indicates that genetic background effects both the response of the animals to α-synuclein expression at the level of gene expression and in terms of the phenotypic consequences. Further, this can be seen at both a local and global level, with effects seen in traits associated with muscles in which α-synuclein is expressed (bagging and thrashing) and traits that represent a global, whole organism, effects (pumping, development rate and lifespan). Our gene expression analysis identifies a range of changes associated with α-synuclein expression. Many of these – particularly those involved in muscle function and in various stress responses – matched expectations given the site of expression and the known effects of α-synuclein aggregation on cellular function. We also identify changes in lipid metabolism (Table 1 and Additional file 4), an important result given that this has been previously linked to α-synuclein pathology in a yeast model [45] and that there is evidence of a direct association between α-synuclein and lipid droplets [46]. Changes in lipid levels of *C. elegans* expressing α-synuclein in the muscles have previously been observed [47], our results therefore provide a set of candidate genes to investigate how α-synuclein expression may mediate lipid metabolism. We also show that such effects can be expected to vary across *C. elegans* genetic backgrounds.

On a phenotypic level we found that, in comparison to the corresponding wild isolates, the α-synuclein expressing lines: developed more slowly; suffered an increased rate of decline in pharyngeal pumping; exhibited a shorter lifespan; and died from matricidal hatching of eggs at an increased rate. Similar phenotypic effects are observed in other *C. elegans* models of protein misfolding disease. For example, transgenic worms expressing polyQ proteins and Aβ both show a significantly shortened lifespan [38, 48]. A reduced lifespan could therefore be a general toxicity phenotype, providing an indirect measure of organismal dysfunction caused by misfolded protein aggregations in the body wall.

Many of the phenotypic changes we see are either only found in wild isolate genetic backgrounds or are more pronounced in these backgrounds (Fig. 5). This demonstrates that there is variation between isolates that appears to differently affect local and global consequences of α-synuclein expression. Previously, only a single *C. elegans* study has directly investigated the effect of genetic background in the context of protein misfolding disease. That study found complex variation in polyQ40 aggregation and toxicity in three wild isolate backgrounds and in a panel of 21 recombinant inbred lines (RILs) [38]. The RIL analysis also showed that the various effects of polyQ40 expression could be uncoupled [38]. In this study, we also show some evidence that natural genetic variation in *C. elegans* can uncouple different effects of α-synuclein expression (Fig. 5), suggesting that this may be a general pattern.

A caveat with this interpretation is however that a range of incompatibilities between alleles from different *C. elegans* genomes have been identified [43, 44] and that the region surrounding the site of the α-synuclein transgene contains a number of mapped quantitative trait loci affecting various life history traits. For example, ILs with introgressed regions of chromosome IV from CB4856 in an N2 genetic background have identified QTLs affecting lifespan and pumping rate that partially overlap our introgression [49]. Similarly, a complex interaction between alleles from the N2 and CB4856 genomes has been found on chromosome IV [44]. However, little is known about the frequency of such synthetic deleterious effects between other genotypes of *C. elegans*. Proteomic analysis of age-dependent changes in protein solubility by Reis-Rodrigues et al. [50] also indicates that the genes encoding proteins that become insoluble with age are enriched for modifiers of lifespan. Additionally, a heat-stress specific QTL for recovery [51] as well as an eQTL-hotspot have been identified on the left arm of chromosome IV (IV: 1.0–2.5 Mb) [36], while a QTL for maternal hatching rate is at the position of the introgression (chromosome IV ~6M) [52]. That such effects might alter disease-related processes complicates the interpretation of both our results here and the previous work of Gidalevitz et al. [38]. To experimentally address this caveat, we introgressed the α-synuclein transgene into a CB4856 genetic background and undertook comparisons with a line that contains a comparable region of the N2 genome, but no transgene, in a CB4856 background. These comparisons indicated that the N2 introgression alone does not recapitulate the phenotypic effects seen in the α-synuclein expressing line (Fig. 6). As these comparisons again show that the expression of α-synuclein in a CB4856 genetic background results in different and more severe effects than those seen in an N2 background, this strongly supports the view that genetic background needs to be considered more generally when *C. elegans* is used as a disease model. A move to the use of more defined modifications in a range of genetic backgrounds that limited the introduction of other alleles – perhaps via the use of CRISPR to introduce the relevant transgenes – would therefore be ideal. It would also be of interest to determine the extent to which genotype-specific were observed in other models, for example where α-synuclein is expressed in neurons [45]. As there is extensive variation between isolates of *C. elegans* in lifespan and in various stress responses and in lifespan, and that variation has now been identified for two protein misfolding diseases such a development would facilitate the systematic analysis of the role of natural variation in such diseases.

## Methods

### *C. elegans* maintenance and growth conditions

Worms were maintained at 20°C on nematode growth medium (NGM) plates seeded with *Escherichia coli* OP50 [53] and all assays were undertaken at 20°C. Assays were initiated using eggs isolated from gravid adults treated with sodium hypochlorite and NaOH [54]. For the microarray analysis of gene expression and for the pharyngeal pumping assays, these synchronized eggs were allowed to hatch on NGM plates seeded with *E. coli* overnight at 20°C, i.e. these worms were not arrested at the L1 stage. After 48 h, worms in all strains had reached the L4 stage. For the lifespan, development, and thrashing activity assays, the synchronized eggs were hatched on NGM plates without *E.coli*, in order to obtain synchronized L1 larvae. When they reach L4 stage, these assay were then measured or setup will all lines.

### *C. elegans* lines

The *C. elegans* wild types JU1511, JU1926, JU1931, JU1941, CB4856 (Hawaii), canonical strain N2 (Bristol), and the transgenic strain NL5901, which contains the *pkIs2386* transgene [unc-54p::α-synuclein::YFP + unc-119(+)], were obtained from the *Caenorhabditis* Genetics Center. To introgress the α-synuclein::YFP transgene into the wild backgrounds, NL5901 hermaphrodites were mated with wild type males, then five to ten F1 fluorescent males were mated with wild type hermaphrodites. Fluorescent males were then isolated in the F2 progeny of this cross and used to backcross to the relevant wild type. This backcrossing to the wild type background was repeated until generation F7 was reached. At this point, worms were allowed to self-fertilise to obtain homozygotes lines and then cryopreserved. This crossing design was used to produce the strains SCH1511, SCH1926, SCH1931, SCH1941, and SCH4856, with for example, SCH1511 containing the transgene in a JU1511 background.

Five genetic backgrounds – N2, JU1511, JU1926, JU1931, and JU1941 – were used to investigate the phenotypic and genomic effects of α-synuclein expression. SCH4856 was used in separate assays to test the effect of N2 alleles surrounding the transgene integration site. Here, comparisons were made to the CBN093 strain which contains a comparable region of the N2 genome introgressed into CB4856, but no transgene. CBN093 was constructed by back-crossing WN071 [55] with CB4856. The strain was back-crossed followed by segregation for 11 generations until a single homozygous region on chromosome IV was obtained. Thereafter, the strain was inbred for 11 generations and the genotype confirmed by sequencing (SRP154243; https://www.ncbi.nlm.nih.gov/sra) using the approach [56, 57].

### IL genotyping

#### PCR-based genotyping

DNA was isolated from the lysates of 5 individual adults from each line, and then was used for genotyping PCRs. Genotyping primers utilized insertions/deletions between the CB4856 and N2 genomes, with 41 primer pairs covering the genome used (see Additional file 1 for details of primers and marker locations) [57]. PCR was carried out with the GoTaq DNA polymerase kit (Promega) according to the manufacturer’s recommendations. The sizes of amplified products were assessed by electrophoresis in 1.5% agarose gels stained with Ethidium Bromide. This allowed the size of the introgressions to be determined, with these regions shown in Additional file 1.

#### Hybridization markers

Marker genes found by DNA hybridizations in Volkers et al. 2013 [35] were used to find the genomic position of the α-synuclein introgression in each genetic background. We tested which genomic region was missing markers for the wildtype background, this region then must be from the NL5901 background.

### Sample preparations and RNA microarray analysis

#### mRNA microarrays

In total three independent replicates of the four JU strains and their corresponding transgenic ILs, as well as N2 and NL5901, were analysed. Worms were assayed at 48 h from egg isolation (see details above) and all strains were at the L4 stage. Worms were therefore the same chronological age, and at the same larval stage, but this design would allow the detection of effects of genetic background, α-synuclein expression and the interaction of these effects on development. At this point worms were generated by hatching alkaline hypochlorite-purified eggs and then harvested by centrifugation, washed with M9 buffer, frozen in liquid N2, and stored at − 80 °C until use. The Maxwell® 16 Tissue LEV Total RNA Purification Kit was used for mRNA isolation, following the manufacturer’s protocol with a modified lysis step. In addition to the lysis buffer, proteinase K was added and the sample was incubated at 65°C while shaking at 1000 rpm for 10 min. Thereafter the standard protocol was followed. PolyA RNA was used to generated Cy3 and Cy5-labeled cRNA samples, which were then hybridized to 4X44K slides V2 (Agilent) *C. elegans* whole genome GeneChips, processed, and scanned (full microarray data in ArrayExpress with accession E-MTAB-6960). RNA microarray statistical analysis and data processing were performed using the Limma package for the R software environment [58]. To find the genes affected by α-synuclein, genotype and age, these terms were used as explanatory factors in a linear model (gene expression ~ age + aS * genotype). Significance thresholds were determined by permutations of all spots on the array. In the permutations, the RNA hybridization intensities were randomly distributed over the genotypes and batches. Therefore, the *p*-value that gave a min ratio of false positives/true positives of 0.05 (= − log10(p) > 3.4) was set for convenience, i.e. an FDR at 0.0186 for α-synuclein effect, 0.0249 for genotype effect and 0.073 for the interaction between α-synuclein and genotype.

#### Enrichment

Enrichments were done on genes groups divined by their significance from the linear model a -log10(p) > 2 was used, then a hyper geometric test in R was used to test each GO term for enrichment.

#### PCA

To partition the variation in gene expression a PCA was done on the transcription profiles (the log2 ratios with the mean) of all samples. The first two axis were used for visualisation.

#### Age estimation

Age estimation of the worms sampled for transcriptomics was done by comparing the class I age responsive genes from Snoek et al. [41], as done by van der Bent et al. [42] and Jovic et al. [59]. These genes show a linear increase during development between 46 and 54 h and can therefore be used to estimate the relative differences in development between samples/populations by their transcriptomes. The α-synuclein expressing SCH lines developed more slowly than the JU lines and none of the lines had started moulting at 48 h, so there were no adults on the plates.

### Phenotypic assays

#### Lifespan

Nematodes were cultured on OP50 bacteria without fluorouracil deoxyribose (FUdR) from synchronised L1 juveniles until young adults. When setting up the experiment, in total 100 worms per strain were randomly selected from the population and transferred onto 10 plates (i.e. each plate contains 10 worms). Then, they were transferred away from their progeny every day during the vigorous reproductive period and every other day during the reduced reproductive period. From the young adult stage, worms were examined for signs of life daily. Individuals that were not moving or twitching after gentle stimulation, followed by vigorous stimulation, or that did not exhibit pharyngeal pumping for 30 s, were considered dead. Individual worms that had died from internal hatching of progeny (bagging), or that had crawled off the plates, were censored from lifespan result but were used for maternal hatching analysis. Two biological replicates were done for lifespan assay. Data analysis was performed in R using the ‘survival’ package. The rates of bagging from these assays were also compared to determine if this was affected by α-synuclein expression.

#### Development time

Synchronised L1 juveniles were obtained by allowing eggs to hatch in M9 buffer after three washes in M9 after hypochlorite treatment. Arrested L1s were then transferred to NGM plates, which fed with *E. coli* OP50 and were incubated at 20°C. Tracking observations and inspections were done at regular time intervals. Development time was defined as the period between worm inoculation and the moment at which the first appearance of eggs and the period until the reproductions reach peak level. Three biological replicates were done for this assay.

#### Pharyngeal pumping

To avoid the effect of short term starvation after inoculation on pharyngeal pumping rate, synchronized eggs, isolated as described above, were allowed to hatch on NGM plates with *E.coli* (t = 0 h). Individual worms were then isolated and observed at the L4 stage (t = 48 h, day 2) and as young adults (t = 72 h, day 3). The number of contractions in the terminal bulb of pharynx was counted for 30 s (*n* = 19 per genotype) for the L4s, and for 60 s for 3 day-old worms (*n* = 30 per genotype). Three biological replicates were done for this assay.

#### Thrashing

Synchronized starved L1 juveniles were inoculated on NGM plates with *E.coli* at 20°C. When N2 worms were observed to have reached the L4 stage, worms were transferred into the wells of a 96-well-plate, where each well contained 50μl of M9 buffer [54]. Three biological replicates were undertaken, with 10 worms per well for 3–5 wells per genotype in each of the replicates. Activity was then measured in an 30 min period using a WMicroTracker (PhylumTech). Here, worm movements in the wells are detected as they interfere with an array of micro beams of infrared light and the number of interference events is counted – hence a higher score represents more activity.

#### Statistical analysis

Phenotypic differences between the lines were tested using an ANOVA (model: phenotype~ aS * GB) to test for overall effects and Tukey HSD to test for lines specific differences. Survival curves were tested by log-rank test from the “survival” package in R.

## Additional files

Additional file 1: Markers used in the genotyping of the α-synuclein ILs. (XLSX 22 kb)

Additional file 2: A figure of gene expression markers across the genome. Determination of the α-synuclein introgression in the four wild isolate backgrounds. Marker genes with different expression between N2 and the wild isolates (All) were used to detect the α-synuclein introgression and N2 border regions. The marker genes missing in the α-synuclein lines indicate the N2 border regions and position of the α-synuclein introgression (aS Missing). Different genetic backgrounds are indicated by the different colours. The position(s) where all lines have missing markers show the likely α-synuclein locus, the extra missing markers on chromosome V show a possible extra introgression in SCH1931. (DOCX 201 kb)

Additional file 3: A matrix containing the microarray data and significance values from the linear model (TXT 3344 kb)

Additional file 4: Gene ontology analysis (GO) of genes identified as affected by Age, Genotype (GB), α-synuclein (aS) and the interaction between genotype and aS, and the details of genes shown in Fig. 1b including the *p* values and average log2 ratios with the mean per genotype for the 78 genes highly specific for aS relative to both Age and GB. (XLSX 246 kb)

Additional file 5: Lifespan assays in α-synuclein ILs and relevant wild isolates. (TXT 2 kb)

Additional file 6: Rates of matricidal hatching in α-synuclein ILs and relevant wild isolates. (TXT 574 bytes)

## Abbreviations

AD: Alzheimer’s disease
ANOVA: Analysis of variance
aS: α-synuclein
ATP: Adenosine triphosphate
Aβ: Amyloid-β
CGC: *Caenorhabditis* Genetics Center
CRISPR: Clustered regularly interspaced short palindromic repeats
eQTLs: expression quantitative trait loci
F2 (number *i.*): Second (*i.* th) filial generation
FDR: False discovery rate
FUdR: Fluorouracil deoxyribose
GB: Genotype
GO: Gene ontology analysis
GWAS: Genome-wide association studies
IGF: Insulin/insulin-like growth factor
ILs: Introgression lines
L1/L4: Larval stage 1/4
Mbp: A megabase pair
NaOH: Sodium hydroxide
NGM: Nematode growth medium
PCA: Principal component analysis
PCR: Polymerase chain reaction
PD: Parkinson’s disease
polyQ: polyglutamine
QTL: Quantitative trait locus
RILs: Recombinant inbred lines
Tukey HSD: Tukey’s range test honestly significant difference
WT: Wild type
YFP: Yellow fluorescent protein
