# Supplementary material for: Genetic background modifies phenotypic and transcriptional responses in a C. elegans model of α-synuclein toxicity
Source: BMC Genomics. 2019 Mar 20;20:232. doi: 10.1186/s12864-019-5597-1 (PMC6427842; doi:10.1186/s12864-019-5597-1)
Supplement: Supplementary file 2 — A figure of gene expression markers across the genome. Determination of the α-synuclein introgression in the four wild isolate backgrounds. Marker genes with different expression between N2 and the wild isolates (All) were used to detect the α-synuclein introgression and N2 border regions. The marker genes missing in the α-synuclein lines indicate the N2 border regions and position of the α-synuclein introgression (aS Missing). Different genetic backgrounds are indicated by the different colours. The position(s) where all lines have missing markers show the likely α-synuclein locus, the extra missing markers on chromosome V show a possible extra introgression in SCH1931. (DOCX 201 kb) [file 12864_2019_5597_MOESM2_ESM.docx]

|  |
| --- |
| Figure S1: Gene expression markers across the genome. Determination of the α-synuclein introgression in the four wild isolate backgrounds. Marker genes with different expression between N2 and the wild isolates (All) were used to detect the α-synuclein introgression and N2 border regions. The marker genes missing in the α-synuclein lines indicate the N2 border regions and position of the α-synuclein introgression (α-synuclein Missing). Different genetic backgrounds are indicated by the different colours. The position(s) where all lines have missing markers show the likely α-synuclein locus, the extra missing markers on chromosome V show a possible extra introgression in SCH1931. |
